# Improving continuing medical education by enhancing interactivity: lessons from Iran

**Published:** 2016-04

**Authors:** SEYED ALIAKBAR FAGHIHI, HAMID REZA KHANKEH, SEYED JALIL HOSSEINI, SEYED KAMRAN SOLTANI ARABSHAHI, ZAHRA FAGHIH, SAGAR V. PARIKH, MANDANA SHIRAZI

**Affiliations:** 1 Department of Medical Education, Iran University of Medical Sciences, Tehran, Iran;; 2 Department of Health in Emergency and Disaster, University of Social Welfare and Rehabilitation Sciences, Tehran, Iran;; 3 Department of Clinical Science and Education, Karolinska Institute, Stockholm, Sweden;; 4 Infertility & Reproductive Health Research Center, Shahid Beheshti University of Medical Sciences, Tehran, Iran;; 5 Department of Medical Education, Faculty of Medicine, Iran University of Medical Sciences, Tehran, Iran;; 6 Shiraz Institute for Cancer Research, School of Medicine, Shiraz University of Medical Sciences, Shiraz, Iran;; 7 University of Toronto, University Health Network, Canadian Network for Mood and Anxiety Treatments, Toronto Western Hospital, Toronto, ON, Canada;; 8 Research Deputy of Medical Education Department, Tehran University of Medical Sciences, Tehran, Iran

**Keywords:** Continuing medical education, General practitioners, Qualitative study

## Abstract

**Introduction:**

Continuing Medical Education (CME) has been considered as a lifelong commitment for doctors to provide the optimal care for patients. Despite a long history of creating CME programs, outcomes are far from ideal. The present qualitative study aims to clarify the barriers affecting effectiveness of the CME programs in Iran based on the experiences of general practitioners.

**Methods:**

Sixteen general practitioners were recruited to participate in in-depth interviews and field observations concerning experiences with CME. The study was performed using a qualitative content analysis method. The codes, categories and themes were explored through an inductive process in which the researchers moved from specific to general.

**Results:**

The participants’ experiences identified a number of barriers, particularly insufficient interaction with the instructors; additional problems included the teachers’ use of an undifferentiated approach; unreal and abstract CME; and ignorance of the diverse reasons to participate in CME.

**Conclusion:**

Based on the study results, there are multiple barriers to effective implementation of CME in Iran. The key barriers include insufficient interaction between the trainees and providers, which must be considered by other stakeholders and program designers. Such interactions would facilitate improved program design, invite more specific tailoring of the education to the participants, allow for more effective educational methods and set the stage for outcome evaluation from the learners actually applying their new knowledge in practice. Replication of these findings with another sample would improve confidence in these recommendations, but these findings are broadly consistent with findings in the educational literature on improving the efficacy of CME.

## Introduction

The emergence of new information and technologies has necessitated medical sciences to experience constant change so that the doctors need ongoing education to be up-to-date and to provide optimal care for their patients (1, 2). Continuing Medical Education (CME), as a part of a developed system of worldwide health services, has been approved as an approach for changing the behavior of physicians in solving diagnostic and medical problems within health systems (3). Developing both quantity and quality in CME has long been a focus of close attention for academic centers and accreditation institutes (4, 5).

For the individual physician, participating in CME is considered as one of the fundamentals of medical professionalism, requiring a lifelong commitment (6). Despite setting broad goals for CME to bring doctors’ clinical knowledge up to date, numerous medical errors are still observed, which is far from the desired outcomes anticipated from CME in the practice (7). Medical education experts believe that the effectiveness of CME in doctors’ changing behaviors could be achieved through interaction in continuing education activities. Compared with the traditional lecture-based methods, interactive approaches were actually more effective in conveying new information and making changes in doctors’ behavior (5, 8-9). However, because of the ease, lower cost, and familiarity of holding traditional lecture-format CME events, most programs are currently held in the traditional manner where interactivity is limited (10).

CME programs in Iran are compulsory and usually involve traditional approaches (11, 12). Once graduating from medical schools after seven years, general practitioners work in different fields such as clinics, hospitals, emergency rooms, and medical health networks. They must gain 125 CME credit points for a period of five years, 25 points per year, to be able to continue their careers, which can be obtained in different ways (13). Studies have identified, in a preliminary way, various problems with Iranian CME activities: lack of coordination between the programs and the field of activity; insufficient attention to professional demands and the educational content; inappropriate teaching styles; and ineffective programs (13-17). According to our knowledge, most studies conducted in Iran related to CME have quantitative approaches; a few qualitative studies can be found to report the barriers on the effectiveness of CME programs. In response, the present study was designed to clarify the barriers affecting effectiveness of the CME programs in Iran, based on experiences and perceptions of general practitioners.

## Methods

The present study was conducted in Fars province, which is home to one of the major medical educational centers in south of Iran. Educational institutions in Shiraz, the capital of Fars Province, have major responsibility for training expert personnel for health who provide treatment both in Shiraz and neighbor towns including Jahrom. The study participants were selected using the purposive sampling method. The inclusion criteria was participating in CME programs on a permanent basis, with maximum variety in terms of workfields  such as in clinics, hospitals, Emergency Rooms (ER), and medical health networks. Accordingly, interviews were conducted with14 male and 2 female general practitioners with an age range of 30 to 58 (mean= 43.4 years) and an average of 14.8 years of employment (Table 1).

**Table 1:** Participitants’ characteristics

** Participants(1) **	**Age (years)**	**Gender**	**Working history (years)**	**Working fields**
**1 **	58	Male	28	Clinics
**2**	36	Male	7	Clinics
**3**	55	Male	26	Clinics
**4**	30	Female	3	Hospitals
**5**	44	Male	18	Clinics
**6**	42	Male	12	Emergency rooms
**7**	43	Male	8	Clinics
**8**	44	Male	14	Health networks
**9**	38	Male	11	Health networks
**10**	41	Male	15	Clinics
**11**	44	Male	18	Emergency rooms
**12**	43	Female	12	Clinics
**13**	44	Male	18	Hospitals
**14** **15** **16**	44 43 45	Male Male Male	18 12 17	Clinics Health networks Emergency rooms

^1^Participitants in this study were general practitioners.

The data of the study were directly collected from the experiences of the study participants. The codes, categories and themes were explored through an inductive process, in which the researchers moved from specific to general. The consequently formulated concepts or categories were representative of the participants’ experiences. The researcher participated in some traditional CME programs, being a firsthand witness to the interaction between participants, instructors, and program providers. The data were collected from 2013 to 2014 for two years. In the beginning, semi-structured, in-depth interviews as well as field notes were used as the strategy of data gathering. The purposive sampling continued until the saturation was reached, i.e. no further information was obtained for the resulted concept. Each interview took an average time of 45 minutes, which was carried out by the first author in Persian, and was literally transcribed. Content analysis was done based on the Persian data prior to translation into English. The interview guide included a short list of general questions e.g. “How do you describe your recent experience of taking part in a CME program?” and “What kind of problems you experienced during CME program?” The questions were followed with respect to the subjects’ experiences in CME; for instance, they were asked to give more examples or clarify their reasons to describe the topic mentioned in the question. The interviews were listened by the principal investigator for several times until an overall idea was obtained and ambiguities were resolved by checking the transcript data with participants soon after the interview or simultaneous at the time of interview. Then, the meaning units and parts of data, which were directly related to the research question, were identified. Subsequently, the data analysis was performed based on Graneheim et al. (18). To find similarities and differences, the preliminary codes were read for several times and compared. Finally, the categories and subcategories were developed and integrated to the themes.

**Rigor**

The credibility of the data was established by the triangulation strategy, which uses a specialized team combination to examine the findings. Besides semi-structured interviews, note taking during interviews and prolonged engagement with data during the past two years were used for data credibility (19). Moreover, the data dependability was assessed, using both peer-and member-checking (20). The primary findings of the study along with the preliminary codes and categories were presented to some of the participants, and their opinions were received (member checking). Some parts of the data were analyzed by other colleagues who were not involved in the study (peer checking) in which the similarities with the current analysis came into view (18). In addition, the findings were repeatedly assessed and checked by supervisors (expert checking). The evidence from other studies, ideas by other researchers and documentation of the study helped to improve the confirmability (21). Finally, by providing a comprehensive description of the topics, participants, data collection and analysis procedures, and limitations of the study, we hope to have created transferability so that other researchers may clearly follow the research process taken by the researchers (19-20).

**Ethical approval**

This study was approved by the Ethics Committee of Iran University of Medical Sciences (ref. no. 94/d/105/474) and informed consent was taken from all participants. At the beginning of the interview, the participants were acquainted with the purpose of the study, method of interviewing, privacy, and confidentiality of the study. The interviews were done individually, all in the places suggested by the interviewees such as a hospital or clinic at an agreed time. The interviewees were allowed to leave the study at any possible time. 

## Results

In the present study, regarding the barriers influencing the effectiveness of CME programs, the experiences and perceptions of general practitioners were explored. As presented in Table 2, according to data analysis, four themes containing several major categories and codes were extracted including insufficient interaction, undifferentiated approach, unreal and abstract CME, and reasons of participation in CME programs. Each theme is described in more details below.

**Table 2:** Examples of extracting codes, categories and themes from raw data

**Meaning unit**	Code	Category	Theme
“Retraining program is to promote the doctors; when he/she is not considered, when their opinions and suggestions are not mentioned, when their requests and ideas do not put into the practice, surely it won’t be ever successful.” “I’ve never seen a university sends forms to ask what programs you’d like us to hold for you.”	Lack of attention to doctors’ views and suggestions prior to designing the program; Lack of interaction with regard to choice of topics; Lack of compilation of doctors’ views and reflecting them in the program Not communicating with doctors prior to program.	Insufficient interaction with trainees prior to planning the programs	Insufficient interaction in CME programs
“The instructor would go there, without attracting attention, very strict. He didn’t care about the audience, just showing his slides. Maybe in around 80% of sessions we attended, we were just listeners”.	Instructors’ inattention to the audience while instructing; Instructors’ Lack of communication with the audience	Insufficient interaction between instructors and trainees during the course of program	
“It might be the case that a participant, twenty years older than me, asks some questions that I might find them funny, or vice versa, I ask some questions that he might find them funny because of having more experiences.” “In a lecture with 100 to 200 attendants, the audience had the feeling no one could interact with that doctor or lecturer.”	Heterogeneous mass hindered communication between instructors and trainers		
“Doctor, wish we had an archive system; wish we collected doctors’ prescriptions, so that my prescription from 20 years ago could be compared with that of the present to tell me what I’ve done. I wish I’d received feedback, and I’d change my behavior better accordingly.	Lack of giving feedback after the program	Lack of interaction after executing or implementing the programs	
“They should take exams, should not to leave us on our own, or we will be illiterate”	Need for evaluating the doctors after the program		
“In CME the trainees are left on their own; it does not bring about durability needed to update doctors. You get acquainted, then you’re on your own till the next program. Because you won’t get a chance to use it now, the use is made for couple of months later. CME should be the way that evaluates my performance."	Leaving the doctors on their own after the program until further notice not creating durability needed to update doctors; doctors’ reluctance to utilize the content after the program		
“A director of an insurance company takes a seat here, a doctor with an office comes; family physician comes; this is just a heterogeneous environment. All have to learn the same thing. Every physician should be trained in his/her own field of work.”	Providing content regardless to individual differences and fields of work	Presenting the same content for everyone	Undifferentiated approach in CME programs
“Just because of being a general practitioner, one can join in all types of a relevant program, even though being not related to their own work fields.”	Administrators’ lack of criteria for participating of learners	Undifferentiated approach recruiting trainees regardless of their work	
“Early on, there used to be classes. Yet, even since then the debate was whether 25 annual credits is really enough for a doctor whose knowledge goes back to 20, 30 years ago, or for a doctor just graduated a year ago. Are they the same? Well, looks like they failed to some extent to get it trough.	Making no distinction for doctors in designing a program since the beginning of program holding nationwide	Lack of a special or tailored approach in educational design	
“Most of CME programs I’ve taken part in by now were all lecture-based. Look, we are a bunch of people with different needs, different interests in learning; but in consecutive years, only a specific and similar method by the presenters …this exactly cannot be matched. This just reduces the efficiency of the program.”	Presenting programs with the same method in consecutive years	Lack of a special or tailored approach in educational instruction	
“There was an educational seminar on crisis management. Once I entered the hall, I saw a dentist, a general doctor, a midwife, a nurse … being there. I thought that might God save the speakers’ soul. What is he going to say? Each of these folks has their own positions.”	Holding combined programs participated by various groups; making no distinction between those groups in terms of experience, scientific level, and working status in the program		
“When a doctor is in the society, they are facing familial, economic, cultural, social, and many other issues. You can’t go and teach them separately.”	Ignoring non educational issues in designing the program	Non comprehensive educational design	
“…The doctor feels whether he takes part or not he can keep going his own business.”	Feeling of detachment between doctors’ performance and the program	Inconsistency between CME and Doctors professional needs	Unreal and abstract CME
“…It is only effective in extending their license for practice and nowhere else, neither in their work or their vision.”	Ineffectiveness in their job, vision, and professional destiny		
“Apparently, the whole debate was a theoretical one instead of being practical to let the trainee learners in practice.”	Offering theoretical and unpractical instruction	Inapplicability in clinical practice	
“Well, the slides weren’t actually applicable in the doctor’s office. You could see that’s the mentioned case, yet they never fully explained its management.”	Insufficient issue instruction. not being practice guiding in doctors’ offices		
“Now, what I know is performed like a cliché. The instructors speak of their own points. We’re not lacking in terms of content and richness of material presented. But is that really of any use to a general practitioner. We used to view it with suspicion.”	Stereotypical style of conducting the program Doubt in applicability of the content for general practitioners		
“One of the most discussed matters is that friendly get-together of doctors and friends see each other”	Contribution to social interactions between doctors as a participating motivation	Motivating factors	Reasons to participate in CME
“Most of the friends and colleagues with office only have eyes for the scores.” “First of all, to tell my interests: I’m personally interested in participating.”	Getting credits as a motivation for participating Personal interest as an impetus for participation		
“I don’t take part for the sake of scores; I do it for scientific aspects.”	Scientific aspect highlighted as a motivation for participation		
“Look, the problem with the program is its compulsory nature. I’m obliged to credit 25 each year to extend my license. I think, based on discussions I had with my friends, this is somewhat discouraging among on our colleagues.”	Compulsory nature of the programs serving as the effective factor for participating	Legal framework	
“Our trainee looks on CME more as a legal requirement than as an actual feeling of need. I mean I have to take the course at long last if I’m to practice. There is no conviction that taking it is beneficial or enabling.”	Feeling no real need for participation; taking part based on legal requirement; obligatory participation to be authorized to practice		

**Insufficient interaction in CME programs**

The participants’ experiences indicated that there were no enough interactions among the trainers, the trainees, and the providers in CME programs. This concept, which was frequently observed in our results, containing three more related sub-concepts including providers’ insufficient interactions with trainees prior to the program planning, insufficient interactions between the instructors and trainees during the training program, and lack of interaction after executing or implementing the programs.

**Insufficient interaction with trainees prior to planning the program**

Based on experiences of the participants, the doctors, as major audiences, not only were excluded from CME program development, but also their opinions and suggestions were not considered. In fact, there was no collaboration between the participants and the providers which reduces the effectiveness of the CME program 

For instance, participant No. 14 said that “*Retraining program is to promote the doctors; when he/she is not considered, when their opinions and suggestions are not mentioned, when their requests and ideas won’t be put into the practice, surely it won’t be ever successful.”*

**Insufficient interaction between instructors and trainees during the course of program**

In addition to age and gender, the participants in the CME programs were very heterogeneous in terms of professional experiences, work fields, and scientific levels. These disparities result in a reduced interaction between participants and instructors, leaving a deterrent effect on the former. 

Participant No. 4 said,* “It might be the case that a participant, twenty years older than me, asks some questions that I might find them funny, or vice versa, I ask some questions that he might find them funny because of having more experience.”*

On the other hand, passive methods in teaching such as lecturing and one-sided discussion by the instructors usually leave no rooms for questions and answers or further explanations and laying inadequate ground for involvement of trainees in the discussions. 

In line with this, participant No. 9 mentioned, *“Mostly, the workshops and seminars we participated were made up of a group of listeners with just one speaker. At the end, we would ask a couple of questions. It was not like this that someone maneuvered in the middle of the discussion to expand it. That has not been the method up to now.”*

**Lack of interaction after executing or implementing the CME programs**

In the participants’ opinion, carrying out a program without following up and monitoring was not considered as CME. They said that they were left on their own from the end of one program until the next, and no follow up was done by the providers regarding the modifying or revising their clinical performances. They believed that, in the long time, this has led to the outdated knowledge of the more experienced physicians.

*Participant No. 1 said that “We wish we had an archive system, collected doctors’ prescriptions, so that my prescription from 20 years ago could be compared with that of the present to tell me what I’ve done. I wish I’d received feedback, and I’d change my behavior better accordingly.”*

Some participants emphasized the necessity of examining and monitoring the performance to enhance the clinical practice. 

Similarly, participant No.5 added: *“…We’d rather be considered not left on our own. By the end of a seminar, we’d rather not be told to wait until further notice. If they leave us alone, we’ll be illiterate. If you look at veteran doctors, you realize they are well behind the times, knowing only four or five medication items. Nevertheless, if we, doctors, are constantly demanded, we will be brought up more knowledgeable. Anyway, if we take a pretest and to be monitored afterwards, we think it will be much better.”*

**Undifferentiated approach in CME programs**

An undifferentiated CME program was another major barrier derived from our analysis. Based on the experiences and perceptions of participants, the same program with the similar content and methodology, irrespective of individual differences such as work fields, motives, and needs, is designed and conducted to instruct all target groups. This could be described by four relevant sub-concepts: presenting the same content for everyone, undifferentiated approaches in recruiting trainees regardless of their work fields, unique approach in designing a program, and a unique approach in instruction. 

**Presenting the same content for everyone**

The participants believed that the presented contents did not correspond to items such as differences in the work fields, scientific level of the audiences, and professional experiences of the participants, and actually, the same content was presented to everyone in practice. 

Participant No. 3 stated that “*A director of an insurance company takes a seat here, a doctor with an office comes; a family physician comes; this is just a heterogeneous environment. All have to learn the same thing. Every physician should be trained in his/her own field of work.*”

**Undifferentiated approaches in recruiting trainees regardless of their work fields**

Doctors participate in a program just because of being a doctor. In fact, no rules are proposed by the providers for learners’ participation in the program.

This is evident from what participant No. 15 said: *“Just because of being a general practitioner, one can join all types of relevant programs, even though not being related to their own work fields.”*

**Lack of a special or tailored approach in educational design**

According to participants experiences, another problem was provision of integrated programs in which other health professions such as specialists, nurses, midwives, and so on, were present whilst their work status was not taken into account, so the mentioned program was not properly efficient for participants which resulted in their dissatisfaction. 

That is why participant No. 13 stated: *“There was an educational seminar on crisis management. Once I entered the hall, I saw a dentist, a general practitioner, a midwife, a nurse … being there. I thought that might God save the speakers’ soul. What is he going to say? Each of these folks has their own positions.”*

**Lack of a special or tailored approach in educational instruction**

Applying a similar methodology to train various types of CME programs was one particular issue at the center of the most participants’ attentions. In their opinions, the fact that everyone receives training through the same methodology led to ineffectiveness of the programs to a large extent.

Participant No. 2 said: *“Most of CME programs I’ve taken part in by now were all lecture-based. Look, we are a bunch of people with different needs, different interests in learning, but in consecutive years, only a specific and similar method by the presenters …. This just reduces the efficiency of the program.”*

**Unreal and abstract CME**

It was presumed by the participants in the study that the CME was unreal, which denotes its ineffectiveness in both clinical practice and improvement of doctors’ performances. As clarified below, it can be illustrated by three associated sub-concepts: inapplicability in clinical practice; inconsistency between CME and doctor’s professional needs; and non-comprehensive educational design.

**Inapplicability in clinical practice**

Based on the doctors’ experiences, the materials were specialty-based and limited to general issues or, sometimes, outside the general practitioners’ expertise area, and/or not sufficient to resolve the doctors’ forthcoming working problems. 

In connection with this, participant No. 13 mentioned, *“The person who’s come to give a speech, only has given all general and specialized issues, but when it comes to the treatment part, he has not said anything. Forget about the formalities; when a session was over, I said ‘we didn’t get the knack.’”*

**Inconsistency between CME and doctors’ professional needs**

Doctors’ experiences showed that for the time being, CME had no effects on their future work, and they did not believe that it brought any particular capabilities or any improvements in curing the patients. Actually, CME does not go hand in hand with clinical practice, thus remaining detached from their professional needs.

In fact, it is seen in the words of participant no. 6:*“Now we know where the problem is. Now the errors in doctors’ prescriptions are brought into their notice. If now we put the previous formulated blocks from ten years ago, this is abstract. The doctor feels whether he takes part or not; he can keep on going his business.”*

**Non-comprehensive educational design**

Based on their experiences, doctors did not live in a vacuum; their practice is affected by external surroundings including livelihood as well as social, political and cultural pressures, whereas CME merely focuses on clinical issues. In their opinions, this type of education is an abstract, and one-dimensional one. 

Again, participant No. 3 added, *“When a doctor is in the society, they are facing familial, economical, cultural, social, and many other issues. You can’t go and teach them separately.”*

**Reasons to participate in CME**

As mentioned earlier, reasons to participate in CME constituted another concept derived from the analysis. Doctors’ reasons to participate in CME were composed of a spectrum ranging from mere motivation and performance improvement to simply earning the permit to get the credits to extend the relicensure in the fulfillment of legal frameworks. This is delineated by two associated concepts: motivating factors and legal frameworks.

**Motivating factors**

No physician is willing to be in the dark about state of the innovations in the medical sciences. All of them are interested in learning about advances in the area of their expertise.

This brings participant No. 12 to acknowledge that: *“At first, to mention my interest; I was interested to participate. I liked to see what sorts of developments have happened, whether they are new or not. I took part because I was looking forward to this stuff.”*

**Legal frameworks**

One of the issues that the majority of participants laid emphasis on was the compulsory presence in CME to get their licensure, which provoked displeasure and dissatisfaction with the programs and the lack of importance for them. 

For instance, participant No. 16 said that *“Our trainee’s view towards CME is more a legal requirement rather than a feeling of need; I mean that if I want to practice medicine, I have to go through the course. There is no belief that taking the course has any benefits for him or provides an advantage or a capability in any sense.”*

## Discussion

The present study was conducted to explore, based on the general practitioners’ experiences, the barriers of CME programs effectiveness. Results of the study revealed four main concepts including insufficient interaction in CME programs, an undifferentiated approach, unreal and abstract CME, and reasons to participate in CME as the main barriers.

According to the study results, the first and one of the most important barriers affecting the effectiveness of CME programs is insufficient interactions, which has been more explored with some related sub-concepts including insufficient interactions between the trainees and providers before planning the program, during the program, and after carrying out the CME programs. Insufficient interaction refers to inadequate mutual communication or negotiation during planning the programs. Besides little discussion and/or question and answer in the course of the training programs, once the program is finished, the participants are left on their own until the next program.

Gratini, et al. (2011) argue that interaction and applicability are two major factors involved in the effectiveness of CME programs in terms of changing doctors’ behaviors (9). Moreover, Shirazi, et al. (2013) showed that limited interaction as well as inapplicability of the topics with GP’s workplace needs covered in CME programs are the reasons of doctors’ dissatisfaction with the programs (12). Making an effective program begins with involving the target audience in the planning of the CME.  Here, we found that the trainees did not have any collaboration with the providers in designing or planning the programs. In addition, Wisenberg, et al. (2002) stated that there was a specialist vision dominancy through the program planning in designing. In short, the program is teacher-focused rather than student-focused.  In most CME programs, specialists are involved as trainers; the difference between their views in providing the appropriate educational content and what the general practitioners demandleads to more dissatisfaction of the latter with the programs, particularly when no negotiations or collaborations are between the instructors and the trainees (22). However, more studies are needed to investigate the relationship between doctors’ dissatisfaction and reduced effectiveness of the programs. Beyond being excluded from planning, our respondents identified problems at the next level, namely during the CME events themselves, insufficient interaction during instruction means less discussion on the issues and less interchange of clinical experience, which resulted in low program effectiveness. This is consistent with the findings of Davis, et al. (2008) denoting reduced effectiveness of the programs when they are passively conducted (5).

Another relevant concept is lack of interaction between the trainees and providers after the program termination. It means that there is no place for discussion; the participants are left on their own. No evaluations of the trainer’s performance are done, and the trainees are not given any feedback.

The use of an undifferentiated approach is the second major barrier associated with CME programs. An undifferentiated approach involves several aspects: recruiting trainees irrespective of their work fields, presentation of similar content to all trainees, and lack of a special or tailored approach in the educational design and instruction. More explicitly, the participants noted that an undifferentiated approach in CME also refers to ignoring participants’ needs, motivations, and also differences among the individuals as well as work fields. As a result, everyone will be trained in the same way and taught the same subjects despite having different needs and learning styles (1). However, as the present findings indicated, no criteria is applied by the providers for recruiting the participants, and the same content through the same methodology is presented to all trainees regardless of their professional differences and scientific levels. Undoubtedly, presence of a patriarchic approach in the programs planning in the designing of educational materials without inclusion of doctors’ needs and expectations could be a proper explanation for the present findings (23), which in turn, seems to pave the way for insufficient interest of the audiences and render CME unimportant in the trainees’ views. A glance at the literature reveals that the dominant teaching methodology in majority of the CME programs is lecture-based; and even the administrators make no distinctions in giving grades to those paid attention to the programs and those who did not (23-24).

Unreal and abstract CME is another major barrier derived from the present study, encompassing several sub-concepts including inapplicability in clinical practice, non-comprehensive educational design, and inconsistency between CME and doctors’ professional needs. It denotes an in applicable, and non-comprehensive educational design, which has no effects on the professional needs of the doctors. In fact, the predominance of an academic approach and a heavily specialist-oriented presentation in CME precede inconsistency between the presented content and general practitioners’ professional requirements (22). In addition, the educational contents are not useful for general practitioner performances and services, which should be provided for the patients. Therefore, as noted earlier, interaction would be an overriding factor to improve the effectiveness of programs and applicability of the educational content in the practice (9, 12).

Another major concept inferred from the study is the doctors’ reasons to participate in CME programs with two related sub-concepts: motivational factors and legal frameworks. Participants believed that this finding, in particular, refers to the driving force that leads to participation of the trainees and trainers in the program. According to their experiences, individuals with different motives, from learning new issues to getting credit points, take part in the programs. CME is also a place to bring back the physicians together with their friends and exchange their professional experiences. However, due to compulsory nature of CME for licensure purposes, getting credit points seems to be still prioritized over professional exchanges. Davis, et al. (2008), believes that acceptance of CME credits as a legal framework to get the relicensure by accreditation institutes has diverted CME from its proposed aims and reduced its level to the plain credit for the doctors (5). This stands in line with our findings, which demonstrates that getting the credits is the only reason for some physicians to participate in the programs. Another important role for CME, namely improving the socialization and peer interactions between attendees, is not considered as an educational goal for the programs participants. This lack of attention to the social purposes of CME which in turn facilitates learning by peer-to-peer discussion could to some extent reduce program effectiveness (5).

## Conclusion

The present study illustrated the main barriers standing in the way of traditional CME programs’ effectiveness in Iran. One of the leading problems derived from the participants’ experiences was insufficient interactions between trainees and providers in the planning, executing, and after implementation of the programs, all of which contribute to reduced effectiveness of the CME program and presumably reduces the chance for trainees to improve their clinical performance. Therefore, it is suggested that participants should be taken into account in the program by the providers not only in planning but also in teaching phases as active partners. We also need to design the programs that consider the differences among individuals and their work fields. In addition, identifying and responding to non-educational issues governing the performance of the physician can provide the circumstances to tailor CME to their professional needs. Improving clinical performance in practicing physicians in Iran thus requires many stepsincluding involving the learner in the process, using interactivity at all stages, tailoring programs to meet actual clinical/professional needs, and providing evaluation of outcomes of the learning to ensure a healthy feedback cycle in the educational process.

**Strength and limitation**

This is the first qualitative study based on the perceptions and experiences of general practitioners, reporting more comprehensively the barriers to the CME programs effectiveness in Iranian context. Exploring the experience and perception of the people involved in the phenomenon and looking through the eyes of experienced participants can bring something new, and can help us to understand the phenomenon under study. However, this kind of study has some limitations. First, the data were gathered through semi-structured interviews, so the results were more comprehensive and subjective. Second, in this study, we just tried to present the barriers of CME, and the process of CME, itself, was not explored; therefore, a grounded  theory approach is recommended for further exploring of CME process in Iran. 
